# MiR-520d-5p directly targets TWIST1 and downregulates the metastamiR miR-10b

**DOI:** 10.18632/oncotarget.2559

**Published:** 2014-11-07

**Authors:** Pinchas Tsukerman, Rachel Yamin, Einat Seidel, Saleh Khawaled, Dominik Schmiedel, Tomer Bar-Mag, Ofer Mandelboim

**Affiliations:** ^1^ The Lautenberg Center for General and Tumor Immunology, The Hebrew University, The BioMedical Research Institute, Israel Canada, Hadassah Medical School, Jerusalem, Israel

**Keywords:** TWIST1, miR-10b, mir-520d

## Abstract

MicroRNAs are key players in most biological processes. Some microRNAs are involved in the genesis of tumors and are therefore termed oncomiRs, while others, termed metastamiRs, play a significant role in the formation of cancer metastases. Previously, we identified ten different cellular microRNAs that downregulate the expression of MICB, a ligand of the activating NK receptor NKG2D. Interestingly, several of the ten MICB-targeting microRNAs, such as miR-10b, are involved in tumor formation and metastasis. In this work, we identify a complex interplay between these different microRNAs. Specifically, we demonstrate that three of the MICB-targeting microRNAs: miR-20a, miR-17-5p and miR-93, also target the same site in the 3′UTR of TWIST1, a transcription factor implicated in cancer metastasis. Additionally, we show that miR-520d-5p targets a different site in the 3′UTR of TWIST1. We next show that the miR-520d-5p-mediated decrease of TWIST1 expression results in reduced expression of one of its targets, miR-10b, and in the restoration of E-Cadherin expression, which in turn results in reduced cellular motility and invasiveness. Finally, we show that miR-520d-5p leads to reduced proliferation of tumor cells, and that high levels of miR-520d-5p correlate with higher survival rates of cancer patients.

## INTRODUCTION

Tumor metastases, which account for around 90% of cancer-related mortality, form in a complex multistep process that includes cell proliferation, invasion, migration, destruction of the extracellular matrix, immune evasion and finally re-colonization [1]. MicroRNAs (miRNAs) are short non-coding RNAs that regulate the activity of more than 60% of our genes [2, 3]. MiRNAs regulate target transcripts by binding predominantly to the 3′-untranslated region (3′UTR) of the target messenger RNA (mRNA). This leads to translational inhibition, followed by destabilization and degradation of the target mRNA [4]. Increasing evidence indicates that miRNAs are involved in the development of cancer, in tumor metastasis and in immune regulation [5–8]. In cancer, miRNAs can function as classical oncogenes (oncomiRs) or as tumor suppressor genes [6].

Earlier work by our group and by others showed that several tumor-promoting miRNAs are also capable of granting tumor cells immune evasive properties [7, 9, 10]. We showed that miR-17-5p, miR-20, miR106, miR-93, miR-520d-3p, miR-373 and miR-10b directly downregulate the stress-induced ligand MHC class I polypeptide-related sequence B (MICB) [7, 10]. MICB is recognized by an important Natural Killer (NK) cell activating receptor named NKG2D [11]. NK cells triggered by binding of ligands to activating receptors can directly kill hazardous cells, such as cancer cells [12]. Thus, MICB dowregulation protects tumors from NK cell-mediated elimination.

Recently Dong et al. demonstrated that one of the MICB-targeting miRNAs, miR-106b, also targets TWIST1 [13]. TWIST1, a basic helix-loop-helix transcription factor, is upregulated in many types of tumors, such as breast, liver, prostate and pancreatic cancer [14–17]. TWIST1 controls the transcription of multiple targets, one of which is the pro-metastatic miR-10b [18]. Thus, the negative regulation of TWIST1 by miR-106b leads to reduced expression of another MICB-targeting miRNA, miR-10b, which is also one of the most well-known metastamiRs. This led us to investigate whether additional MICB-targeting miRNAs, or related miRNAs, may also target TWIST1 to prevent the expression of miR-10b.

## RESULTS

### Several MICB-targeting miRNAs also target TWIST1

**Figure 1:**
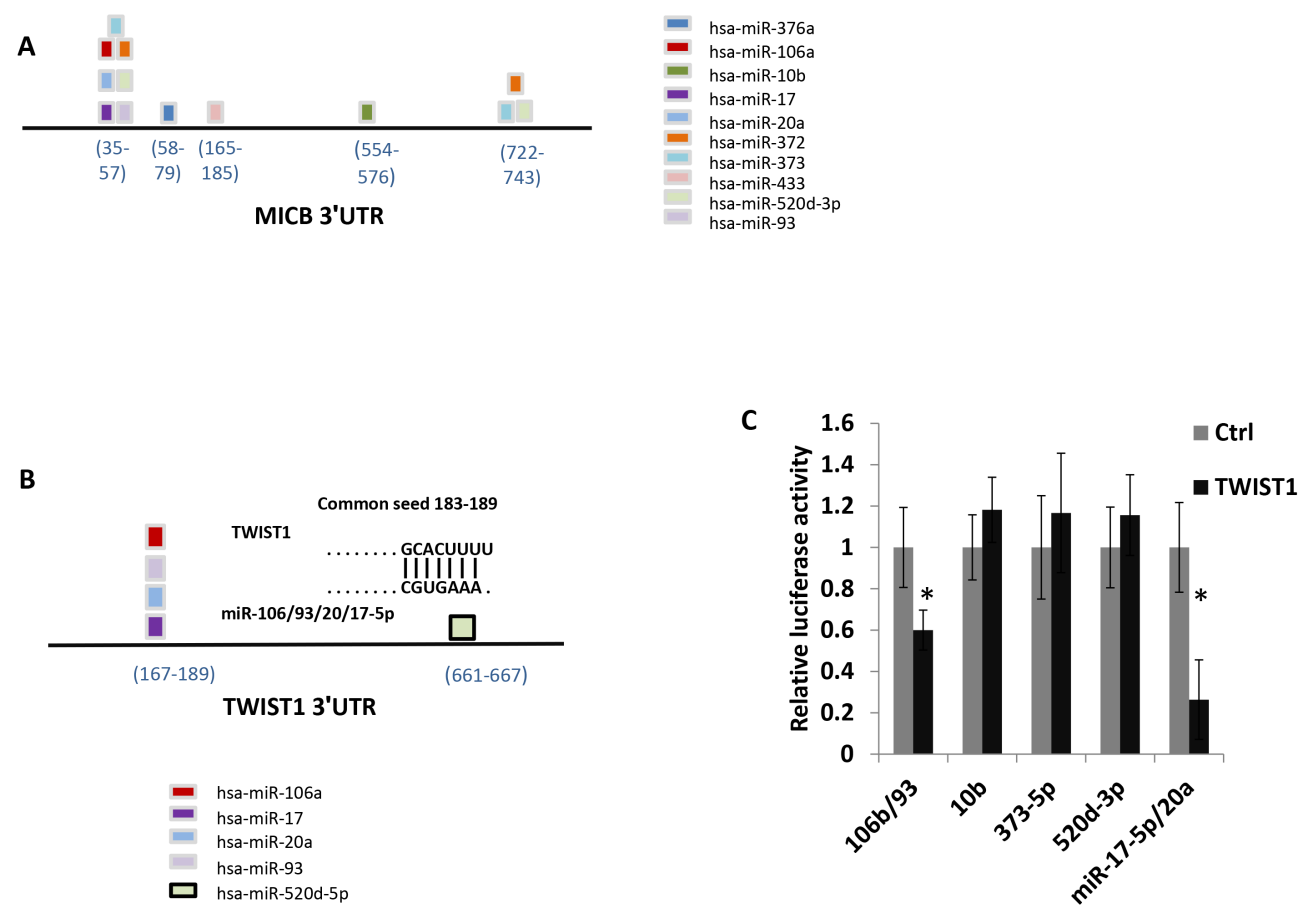
Several MICB targeting miRNAs are predicted to target TWIST1 Schematic representation of MICB 3′UTR including the location of the various MICB targeting miRNAs. **(B)** Schematic representation of TWIST1 3′UTR that includes the location of predicted targeting miRNAs. **(C)** Control 3′UTR, or TWIST1 3′UTR were fused to firefly luciferase, and co-transfected with renilla luciferase into DU 145 cells over expressing the microRNAs indicated at the X axis. miR-17-5p and miR-20a, and miR-93 and miR-106b were co-expressed. The luciferase activity was measured and normalized to renilla activity. The control 3′UTR activity was set to 1. Results are representative of two independent experiments. *P < 0.035.

We have previously demonstrated that MICB is targeted by ten different miRNAs [7, 9, 10]. Some of the MICB-targeting miRNAs bind to two separate sites in the 3′UTR of MICB (miR-372, miR-373 and miR-520d-3p, Figure 1A) while others (miR-93, miR-20, miR-106, miR-17-5p, miR-376a, miR-433 and miR-10b, Figure 1A) have a single binding site in the indicated locations of the 3′UTR. Since it was demonstrated that one of the MICB-targeting miRNAs, miR-106, targets TWIST1 [13] and because TWIST1 controls the expression of miR-10b, a metastamiR that is also involved in the regulation of MICB ([10] and Figure 1), we wondered whether additional MICB-targeting miRNAs might target TWIST1 as well. To investigate this, we used the TargetScan algorithm [2] and RNA-Hybrid software [19] to predict which MICB-targeting microRNAs might target TWIST1. Interestingly, miR-20a, miR-93 and miR-17-5p (which all have identical seed sequence, Fig. 1B) were predicted to target TWIST1 at the same site as miR-106b (seed 183–189), (Figure 1B). To test whether the predicted site in the 3′UTR of TWIST1 is indeed targeted by the relevant MICB-targeting miRNAs, we co-expressed miR-17-5p together with miR-20a, and miR-93 together with miR-106b in DU 145 cells. These miRNAs were co-expressed since they originate from the same polycistronic transcript. We next performed luciferase assays by transfecting the various miRNA-containing DU 145 cells with a luciferase gene fused to the 3′UTR of TWIST1 and assayed the luciferase activity. Importantly, reduced luciferase activity was observed, indicating that miR-106b and other MICB-targeting miRNAs that are predicted to target TWIST1 (miR-17-5p, miR-20a and miR-93) indeed target the 3′UTR of TWIST1. In contrast, miR-10b, miR-520d-3p and miR-373 (which are not predicted to target TWIST1 3′UTR) did not affect luciferase activity (Figure 1C). To validate the specificity of this effect we mutated the predicted shared seed site of miR-106b, miR-17-5p, miR-20a and miR-93 in the 3′UTR of TWIST1. We next used the DU145 cells that co-express miR-93 together with miR-106b (Supplementary Figure 1A), miR-17-5p together with miR-20a (Supplementary Figure 1B), miR-520d-3p (Supplementary Figure 1C) and miR-373 (Supplementary Figure 1D) and transfected all of these cells either with a luciferase gene fused to the 3′UTR of TWIST1 or with the mutated TWIST1 3′UTR. As can be seen, while inhibition of luciferase activity was observed when the wild type TWIST1 3′UTR was expressed in DU 145 cells that co-express miR-93 together with miR-106b and miR-17-5p together with miR-20a (Supplementary Figure 1B), no inhibition was observed when the 3′UTR of TWIST1 was mutated (Supplementary Figure 1A and 1B, respectively). Inhibition of luciferase activity was not observed in DU 145 cells expressing either miR-520d-3p (Supplementary Figure 1C) or miR-373 (Supplementary Figure 1D).

### TWIST1 is downregulated by miR-520d-5p

**Figure 2:**
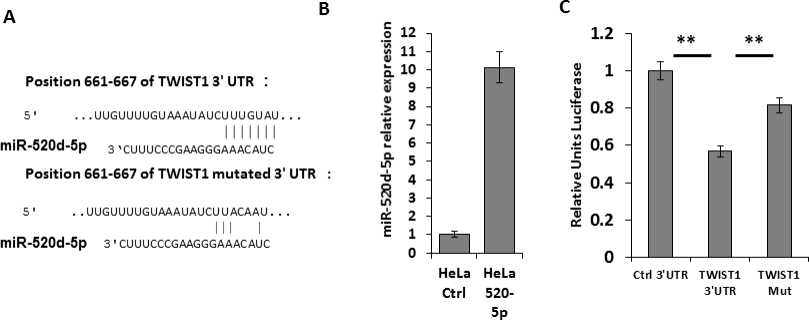
miR-520d-5p targets TWIST1 in its 3′UTR **(A)** Schematic representation of the predicted binding site of miR-520d-5p in the 3′UTR of TWIST1 (upper) and of the mutated site with seed deletion (TWIST1 Mut, lower). The nucleotide positions are indicated **(B)** qRT-PCR for the expression of miR-520d-5p in HeLa cells transduced with either a control miR (CTRL) or miR-520d-5p. **(C)** Control 3′UTR, WT TWIST1 3′UTR or mutated TWIST1 3′UTR (Mut) were fused to firefly luciferase, and co-transfected with renilla luciferase into HeLa cells overexpressing miR-520d-5p. The luciferase activity was measured and normalized to renilla activity. The control 3′UTR activity was set to 1. Results are representative of two independent experiments. **P < 0.015.

Interestingly, miR-520d-5p, which is located at the 5′ end of the stem-loop structure, of the same pre-miRNA which gives rise to the MICB-targeting miRNA miR-520d-3p, was also predicted to have a site in the 3′UTR of TWIST1 (the seed is located in position 661-667, Figure 1B). To investigate whether miR-520d-5p can target TWIST1, we mutated three nucleotides in the predicted binding site of miR-520d-5p (Figure 2A), cloned both the mutated and wild type 3′UTR of TWIST1 downstream to a luciferase reporter gene and tested the luciferase activity in the presence of miR-520d-5p. HeLa cells were transduced with lentiviruses containing either a control miRNA or miR-520d-5p, and the over-expression of miR-520d-5p was validated by qRT-PCR (Figure 2B). The transduced cells were then transfected with the luciferase constructs. As can be seen, in the presence of miR-520d-5p, a reduction of around 50% in luciferase activity was observed when the wild type 3′UTR of TWIST1 was present (Figure 2C). Since mutations in the seed region partially (80%), yet significantly, restored the luciferase activity (Figure 2C), we concluded that the 3′UTR of TWIST1 is directly targeted by miR-520d-5p at the predicted binding site. Similar results were obtained when using MDA-MB-231 cells (data not shown).

**Figure 3:**
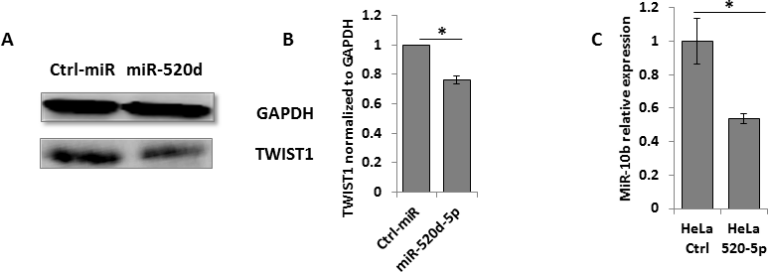
The miR-520d-5p downregulation of TWIST1 leads to decreased miR-10b expression **(A)** Western blot for TWIST1 expression (bottom plots) in HeLa cells over expressing a control miRNA (left) or miR-520d-5p (right). GAPDH was used as loading control (top plots). Quantification of normalized data **(B)** *P < 0.03 results are representative of two independent experiments. **(C)** Levels of miR-10b quantified by qRT-PCR in cells overexpressing miR-520d-5p or control miR (normalized to U6). Results are representative of two independent experiments. *P < 0.03.

To demonstrate that miR-520d-5p can reduce TWIST1 protein expression, we performed western blots for the expression of TWIST1 in the presence of miR-520d-5p or a control miRNA and observed a significant, miR-520d-5p-dependent decrease of TWIST1 expression (Figure 3A, quantified in Figure 3B). Importantly, because TWIST1 positively regulates miR-10b expression [18], we also confirmed that the miR-520d-5p-mediated TWIST1 downregulation resulted in a two-fold reduction in miR-10b expression (Figure 3C).

Next, we wanted to show that the endogenous miR-520d-5p is able to control TWIST1 expression. To this end, we used the miRNA sponge technique which enables effective antagonizing of a specific miRNA [20]. One of the major advantages of the sponge technique is that it also enables monitoring of the sponge activity. The sponge is located downstream to a GFP reporter and thus the sequestration of the relevant miRNA by the sponge leads to reduced GFP intensity.

**Figure 4:**
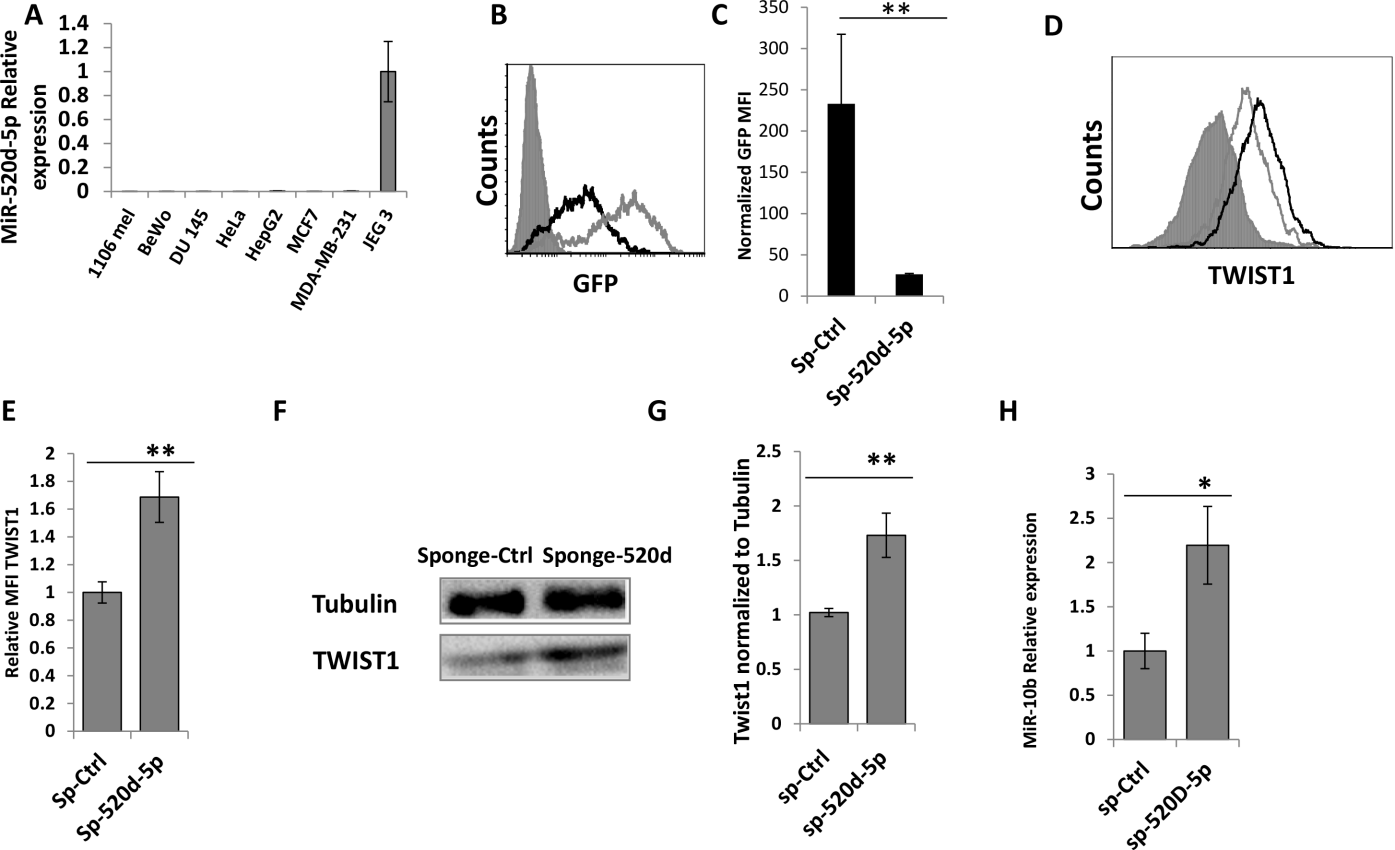
miR-520d-5p endogenously controls TWIST1 expression **(A)** qReal-Time PCR was performed on cDNA extracted from the indicated cell lines (X Axis), for the expression of miR-520d-5p. The data is normalized for U6. **(B)** GFP levels in JEG 3 cells untreated (grey filled histograms), transduced with a control sponge (grey empty histograms) or transduced with anti-miR-520d-5p sponge (black empty histograms). **(C)** Normalized GFP MFI levels of the control sponge compared to anti miR-520d-5p sponge in JEG 3 cells. **(D)** JEG 3 cells transduced with a control sponge (grey empty lines) or with anti-miR-520d-5p sponge (black empty histograms) and were stained for TWIST1. The isotype match control is the grey filled histogram. **(E)** Quantification of the results from (D) error bars derived from standard deviation, results representative of three independent experiments. **P < 0.01. **(F)** JEG 3 cells were transduced with control sponge or with anti-miR-520d-5p sponge. WB was performed with anti-TWIST1 (bottom) and Tubulin served as control (top). **(G)** The quantification for TWIST1 expression from (F). **P < 0.01 **(H)** qRT-PCR for the expression of miR-10b in JEG 3 cells transduced with a control sponge (sp-Ctrl) or with anti-miR-520d-5p sponge (sp-520d-5p).*P < 0.04

We screened multiple human cancer cell lines (as miR-520d-5p is only expressed in higher mammals [21, 22]) for the expression of miR-520d-5p and found that most of them are negative for the expression of miR-520d-5p, with the exception of the JEG 3 choriocarcinoma cell line (Figure 4A and data not shown). Next, anti-miR-520d-5p sponge and a control sponge were generated, cloned into lentivirus vectors and JEG 3 cells that express high levels of miR-520d-5p (Figure 4A) were transduced with these sponges. As can be seen in Figure 4B, the anti-miR-520d-5p sponge probably sequestered miR-520d-5p as the GFP intensity of this sponge was significantly reduced in JEG 3 cells compared with a control sponge (Figure 4B, quantified in Figure 4C). The levels of TWIST1 were simultaneously elevated, as demonstrated by intracellular FACS staining (Figure 4D, quantified in 4E) and by western blot (Figure 4F, quantified in 4G). Finally, we demonstrated that the expression of miR-10b was also elevated; in correlation with the increase in TWIST1 levels (Figure 4H).

### The miR-520d-5p-mediated downregulation of TWIST1 leads to increased E-Cadherin expression

**Figure 5:**
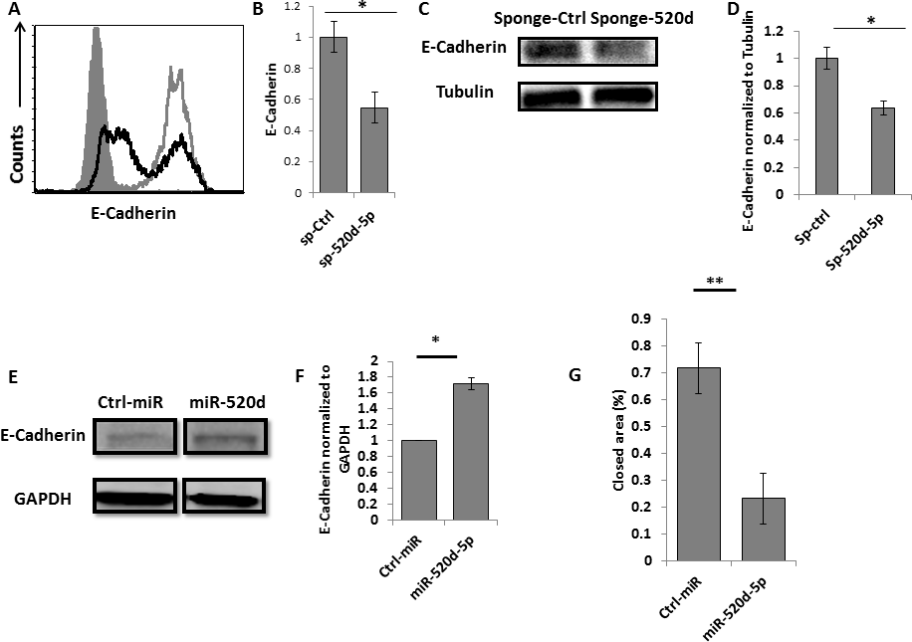
miR-520d-5p affects the levels of E-Cadherin **(A)** Intracellular staining for E-Cadherin in JEG 3 cells transduced with a control sponge (grey empty lines) or with anti-miR-520d-5p sponge (black empty histograms) **(B)** quantification of the E-Cadherin downregulation presented in (A), *P < 0.03 error bars derived from standard deviation, results representative of two independent experiments. **(C)** Western blot for E-Cadherin (top) and Tubulin (control ) bottom plots expression in JEG 3 cells transduced with a control sponge or with anti-miR-520d-5p sponge **(D)** Quantification of the E-Cadherin downregulation presented in (C), **(E)** Western blot for E-Cadherin (top) expression in HeLa cells expressing control miRNA (left) or miR-520d-5p (right). GAPDH was used as loading control (bottom), **(F)** Quantification of the data presented in (E). *P < 0.03 **(G)** HeLa cells overexpressing either control miR or miR-520d-5p were subjected to wound healing assay with images taken at 0 and 16 hours after incubation. The rate of migration was determined by quantifying the total distance that the cells moved from the edge of the scratch toward the center of the scratch. Quantification of the closed area normalized to initial wound distance. *P < 0.02, **P < 0.001. The data represents two independent experiments.

TWIST1 is known to repress E-Cadherin expression [23]. Thus, antagonizing miR-520d-5p, which results in restoration of TWIST1 expression, is expected to reduce the levels of E-Cadherin. Indeed, expressing the miR-520d-5p sponge in JEG 3 cells resulted in a parallel decrease in the expression of E-Cadherin levels (Figure 5A, quantified in 5B). We also validated this result by using western blot assays (Figure 5C quantified in 5D).

Next, we performed the reciprocal experiment and tested whether the miR-520d-5p-mediated downregulation of TWIST1 will result in increased E-Cadherin expression, by testing HeLa cells over-expressing miR-520d-5p. As can be seen, over-expression of miR-520d-5p resulted in significant E-Cadherin upregulation (Figure 5E, quantified in 5F). The upregulation of E-Cadherin is known to reduce migration of various tumors [24]. Indeed, using wound healing assays, we demonstrate, that in the presence of miR-520d-5p, cell migration is significantly impaired (Figure 5G).

### The overexpression of miR-520d-5p results in diminished proliferation and invasiveness of MDA-MB-231 cells

**Figure 6:**
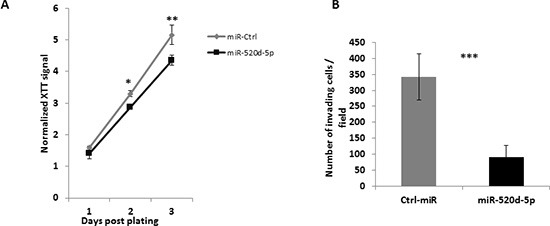
miR-520d-5p reduces proliferation and cellular invasiveness **(A)** MDA-MB-231 cells overexpressing either a control miR (grey line) or miR-520d-5p (black line) were subjected to a XTT assay to measure proliferation rate. Significant differences were observed starting day 2 post plating. P* < 0.03, ** < 0.005 by a two tailed student's t-test. **(B)** MDA-MB-231 cells overexpressing either a control miR (grey bars) or miR-520d-5p (black bars) were tested to their ability to invade through Matrigel covered 8μm pores for six hours. The membranes were washed, fixated and stained, 10 random fields were quantified P*** < 7*10^−5^

The reduction of TWIST1 expression is associated with reduced proliferation and invasiveness of various tumors [25, 26], we thus wanted to see whether the overexpression of miR-520d-5p leads to these effects. We overexpressed either miR-Ctrl or miR-520d-5p in MDA-MB-231 cells and performed XTT assay to assess the effect on cell proliferation. Indeed the overexpression of miR-520d-5p resulted in diminished proliferation of the MDA-MB-231 cells (Figure 6A). We next used those cells in a Matrigel invasion assay and observed that the overexpression of miR-520d-5p strongly inhibits the invasiveness of MDA-MB-231 cells.

### Increased levels of miR-520d-5p correlates with better survival of cancer patients

**Figure 7:**
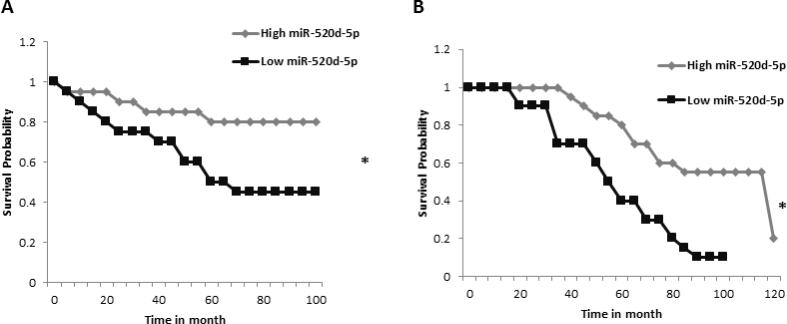
High expression of miR-520d-5p correlates with higher survival percentage Graphical output of the “MIRUMIR” algorithm [27], for the search term “miR-520d-5p” **(A)** Data set for nasopharyngeal carcinoma with the GEO accession GSE36682. **(B)** The data set used for breast cancer with the GEO accession GSE22216. *P < 0.00665.

Finally, we were interested to see what is known as to the clinical importance of miR-520d-5p expression. For that purpose we used the MIRUMIR online tool [27] which performs survival analyses for the input microRNA across multiple expression datasets. We found that high miR-520d-5p expression correlates with higher survival rate of breast cancer patients (“GEO dataset ID: GSE36682”, Figure 7A), and of nasopharyngeal carcinoma patients (“GEO dataset ID: GSE37405”, Figure 7B).

## DISCUSSION

The ability of miRNAs to regulate various aspects of normal development and tumor formation is well-known. With regard to tumors, miRNAs can either promote tumor formation and metastasis, or inhibit these activities, and are accordingly termed oncomiRs/metastamiRs or tumor suppressor miRNAs, respectively. The activity of the oncomiRs/metastamiRs or tumor suppressor miRNAs is regulated by various transcription factors, one of which is TWIST1, which positively regulates the expression of one of the most well-known metastamiRs; miR-10b. MiR-10b is a potent pro-metastatic metastamiR and previous studies have shown that miR-10b targets several tumor suppressors such as, HOXD10 [18], KLF4 [28], TIAM1 [29] and others [30].

We demonstrate here, that miR-520d-5p targets TWIST1 at a specific site located in its 3′UTR. Because mutations in the site targeted by miR-520d-5p did not completely abolish the luciferase inhibition, we suggest that additional miRNAs or RNA binding proteins might also target the 3′UTR of TWIST1. We further demonstrated that miR-520d-5p targeting of TWIST1 resulted in reduced expression of the prominent metastamiR miR-10b. Finally, we showed that the miR-520d-5p-mediated reduction of TWIST1 leads to increase of E-Cadherin expression and to a reduction in cell motility and invasiveness.

We previously demonstrated that miR-10b is not only involved in tumor promotion and metastases, but also in immune escape, as we showed that miR-10b is capable of targeting MICB [10], a stress-induced ligand that is recognized by the potent NK activating receptor NKG2D [11]. Nine additional cellular miRNAs capable of targeting MICB were also discovered by our group [7, 9] and similarly to miR-10b, several of them (such as miR-373 and miR-93) were also shown to be involved in tumor metastases [31, 32]. MICB expression must be tightly controlled. On the one hand, the expression of MICB andother stress induced ligands of NKG2D must be rapidly induced after insults such as viral infection or transformation. On the other hand the expression of the stress induced ligands must be tightly regulated under normal healthy conditions to avoid autoimmunity. MicroRNAs perfectly suitable to achieve this fine-tuning of stress-induced ligand expression and it is therefore not surprising that, as we demonstrate here, some of the ten MICB-targeting miRNAs negatively regulate each other.

We showed that miR-520d-5p, which is derived from the same stem-loop as the MICB-targeting miRNA miR-520d-3p, targets TWIST1, which in turn reduces the expression of miR-10b, which itself targets MICB. It was shown by others that another MICB-targeting miRNA, miR-106b, also targets TWIST1, and here we demonstrated using luciferase reporter assays, that three additional MICB-targeting miRNAs: miR-17, miR-20a and miR-93, also target TWIST1. Hence, four MICB-targeting miRNAs (miR-106b, miR-17-5p, miR-20a and miR-93) and one additional MICB-related miRNA: miR-520d-5p, all target TWIST1, and in turn downregulate the expression of another MICB- targeting miRNA, the metastamiR miR-10b. While we still do not completely understand the reasons for these antagonistic interactions, one possible explanation might be that since miR-10b is such a dangerous miRNA (it is one of the most potent metastasis promoting miRNAs known today, [33]) its expression is tightly controlled even by miRNAs that share some of the activities that are mediated by miR-10b.

Overexpression of miR-520d-5p may be beneficial to cancer patients. First, because, as mentioned above, miR-10b is a potent metastamiR, second, because miR-10b also negatively regulates the expression of MICB, making tumor cells less susceptible to NKG2D-mediated killing [7, 10] and third, because the miR-520d-5p-mediated downregulation of TWIST1 results in restoration of E-Cadherin, and this consequently results in the inhibition of cell motility and invasiveness. MiR-520d-5p upregulation was shown to induce tumor suppressive effects and to inhibit metastasis [34]. It was also suggested that miR-520d-5p might play a role in the conversion of hepatoma tumor cells to benign cells in *in vivo* [35]. Indeed, we show here, that bioinformatics analysis of microRNA expression showed that high expression of miR-520d-5p correlates with better survival. Hence, we suggest that miR-520d-5p might be an attractive candidate for the treatment of developing tumors.

## METHODS

### Cell culture

MDA-MB-231, JEG 3, HeLa and DU 145 cells were maintained in Dulbecco's modified Eagle's medium supplemented with 10% fetal calf serum. All cells were incubated in a humidified atmosphere of 5% CO_2_ and 95% air at 37°C.

### qReal Time PCR

For qRT-PCR analysis of miRNA, cDNA was produced from various cells. Total RNA was isolated using the Quick-RNA™ MiniPrep (ZYMO). All RNAs were polyadenylated with poly (A) polymerase (Ambion). RNA was then reverse-transcribed with Moloney murine leukemia virus reverse transcriptase (Invitrogen) and 0.5 μg poly(T) with adaptor sequence. Reaction primers: reverse primer was a 3′ adapter primer (3′RACE outer primer in the First Choice RLM-RACE kit), and the forward primer was designed based on the entire miRNA sequence. For hsa-miR-10b: 5′-TACCCTGTAGAACCGAATTTGTG 3′, for hsa-miR-520d-3p AAAGTGCTTCTCTTTGGTGGG, hsa-miR-520d-5p CTACAAAGGGAAGCCCTTTC. MiR-16 and U6 snRNA were used as the endogenous reference genes for PCR quantification. The primer and primers based on the microRNA sequences DNA were amplified with specific primers and Platinum SYBR Green qPCR SuperMix-UDG with ROX (Invitrogen) on an ABI PRISM 7900 real-time PCR system (Applied Biosystems).

### Plasmid construction and lentivirus production

Specific oligonucleotides were annealed and inserted into the pTER vector and then were excised from the vector together with the H1 RNA polymerase III promoter into the lentiviral vector SIN18-pRLL-hEFIαp-EGFP-WRPE as described [7]. Lentiviral viruses were produced by transient three-plasmid transfection as described [10]. These viruses were used to transduce HeLa and MDA-MB-231 cells in the presence of polybrene (5μg/ml). Sponge constructs were generated by annealing the oligonucleotides, phosphorylating them using T4 polynucleotide kinase, and inserting them into the pcDNA3 vector (Invitrogen). The sponges were excised and cloned into the lentiviral vector SIN18-pRLL-hEFIap EGFP-WRPE downstream to the GFP cassette [10]. Each sponge consists of 6 adjacent binding sites for the relevant viral miRNA, separated by a 4 nucleotide (AGAG) spacer (Hannon 2008). The sequences of the sponges binding site: sponge anti–hsa-miR-520d-3p: (5′-3′GAAAGGGCAAGCTTTGT); sponge anti–miR-BART 1-5p (control): CACAGCACGTCAGAACACTAAGA

### Luciferase assay

The 3′UTR of TWIST1 was cloned from cDNA extracted from HeLa and JEG 3 cells. The primers for cloning the 3′UTR are: FW GGTCTAGAGCAGGCGGAGCCCCCCAC REV GGTCTAGACTCTAAATTTTTTATATTTATTTATTGC. The inserts and their proper orientation were confirmed by sequencing. For the TWIST1 mutation (miR-520d-5p site), following primers were used: FW 5′ TGTAAATATCTTACAATATTTTTC, REV5′ GAAAAATATTGTAAGATATTTACA. DU 145/HeLa/MDA-MB-231 cells were plated in 24-well plates and 24 h later were transfected with 100 ng of a Firefly luciferase reporter vector and 50 ng of the control Renilla luciferase pRL-CMV (Promega) using the LT1 transfection reagent (Mirus), at a final volume of 0.5 ml. Firefly and Renilla luciferase activities were measured consecutively with the Dual-Luciferase Assay System (Promega), 48 h following transfection. Firefly luciferase activity was normalized to Renilla luciferase activity and then normalized to the average activity of the control reporter.

### Wound-healing assays

HeLa cells were seeded into 12-well plates and allowed to grow to ~95% confluence. A vertical wound was created using a 200 μl pipette tip, and the old medium was replaced with serum-free medium. Images were captured at 0 and 16h under the microscope to assess the rate of gap closure.

### Cell proliferation assays

MDA-MB-231 cells overexpressing control miR or miR-520d-5p were plated in a flat 96 plate at of 2.5*10^3^ per well. The measurements were performed within 24 hour intervals, and were analyzed using an XTT (sodium 3-[1-(phenylaminocarbonyl)- 3,4- tetrazolium]-bis (4-methoxy- 6-nitro) benzene sulfonic acid hydrate) proliferation assay according to the manufacturers instruction (Biological industries, Israel Cat. No.: 20-300-1000).

### Matrigel invasion assay

Blind well chemotaxis chambers with 13 mm-diameter filters were used for this assay. Polyvinylpyrrolidone-free polycarbonate filters, 8 μm pore size (Costar Scientific Co., Cambridge, MA), were coated with basement membrane Matrigel (25μg per filter). MDA-MB-231 cells (2.0 × 10^5^) suspended in DMEM media serum free, containing 0.2% bovine serum albumin (BSA), were added to the upper chamber, over the filter. DMEM containing 10% fetal bovine serum (FBS) was applied as a chemoattractant and placed in the lower compartment of the Boyden chamber. Assays were carried out at 37°C in 5% CO2 for 6 hours. At the end of the incubation, the cells on the upper surface of the filter were removed by wiping with a cotton swab. The filters were fixed and stained with Diff-Quick System (Dade Behring, Inc.). Cells that migrated and invaded through the membrane were counted at ten randomized fields.

### FACS and western blotting

For intracellular staining cells were fixed using BD Cytofix™ fixation buffer and permeabilized using BD Phosflow™ perm buffer I, the monoclonal anti-TWIST1 (Twist2C1a) was used. For isotype match control mIgG1 was used. Western blotting was conducted under standard conditions briefly: Whole cell proteins were separated on 10% sodium dodecyl sulfate–polyacrylamide gel electrophoresis gels and blotted onto nitrocellulose membranes. The filters were hybridized with monoclonal anti-TWIST1 (Twist2C1a), anti-E-Cadherin, Anti-glyceraldehyde 3-phosphate dehydrogenase (used as a loading control) (GAPDH) or anti Tubulin (Santa Cruz Biotechnology) (used as a loading control).

## SUPPLEMENTARY FIGURE



## References

[1] Chaffer CL, Weinberg RA (2011). A perspective on cancer cell metastasis. Science.

[2] Lewis BP, Burge CB, Bartel DP (2005). Conserved seed pairing, often flanked by adenosines, indicates that thousands of human genes are microRNA targets. Cell.

[3] Baek D, Villen J, Shin C, Camargo FD, Gygi SP, Bartel DP (2008). The impact of microRNAs on protein output. Nature.

[4] Guo H, Ingolia NT, Weissman JS, Bartel DP (2010). Mammalian microRNAs predominantly act to decrease target mRNA levels. Nature.

[5] Calin GA, Croce CM (2006). MicroRNA signatures in human cancers. Nat Rev Cancer.

[6] Iorio MV, Croce CM (2012). microRNA involvement in human cancer. Carcinogenesis.

[7] Stern-Ginossar N, Gur C, Biton M, Horwitz E, Elboim M, Stanietsky N, Mandelboim M, Mandelboim O (2008). Human microRNAs regulate stress-induced immune responses mediated by the receptor NKG2D. Nat Immunol.

[8] Tsukerman P, Enk J, Mandelboim O (2013). Metastamir-mediated immune evasion: miR-10b downregulates the stress-induced molecule MICB, hence avoid recognition by NKG2D receptor. Oncoimmunology.

[9] Nachmani D, Lankry D, Wolf DG, Mandelboim O (2010). The human cytomegalovirus microRNA miR-UL112 acts synergistically with a cellular microRNA to escape immune elimination. Nat Immunol.

[10] Tsukerman P, Stern-Ginossar N, Gur C, Glasner A, Nachmani D, Bauman Y, Yamin R, Vitenshtein A, Stanietsky N, Bar-Mag T, Lankry D, Mandelboim O (2012). MiR-10b downregulates the stress-induced cell surface molecule MICB, a critical ligand for cancer cell recognition by natural killer cells. Cancer Res.

[11] Bauer S, Groh V, Wu J, Steinle A, Phillips JH, Lanier LL, Spies T (1999). Activation of NK cells and T cells by NKG2D, a receptor for stress-inducible MICA. Science.

[12] Long EO (2002). Tumor cell recognition by natural killer cells. Seminars in cancer biology.

[13] Dong P, Kaneuchi M, Watari H, Sudo S, Sakuragi N (2013). MicroRNA-106b modulates epithelial-mesenchymal transition by targeting TWIST1 in invasive endometrial cancer cell lines. Mol Carcinog.

[14] Kwok WK, Ling MT, Lee TW, Lau TC, Zhou C, Zhang X, Chua CW, Chan KW, Chan FL, Glackin C, Wong YC, Wang X (2005). Up-regulation of TWIST in prostate cancer and its implication as a therapeutic target. Cancer Res.

[15] Ohuchida K, Mizumoto K, Ohhashi S, Yamaguchi H, Konomi H, Nagai E, Yamaguchi K, Tsuneyoshi M, Tanaka M (2007). Twist, a novel oncogene, is upregulated in pancreatic cancer: clinical implication of Twist expression in pancreatic juice. Int J Cancer.

[16] Sun T, Zhao N, Zhao XL, Gu Q, Zhang SW, Che N, Wang XH, Du J, Liu YX, Sun BC (2010). Expression and functional significance of Twist1 in hepatocellular carcinoma: its role in vasculogenic mimicry. Hepatology.

[17] Yang J, Mani SA, Donaher JL, Ramaswamy S, Itzykson RA, Come C, Savagner P, Gitelman I, Richardson A, Weinberg RA (2004). Twist, a master regulator of morphogenesis, plays an essential role in tumor metastasis. Cell.

[18] Ma L, Teruya-Feldstein J, Weinberg RA (2007). Tumour invasion and metastasis initiated by microRNA-10b in breast cancer. Nature.

[19] Rehmsmeier M, Steffen P, Hochsmann M, Giegerich R (2004). Fast and effective prediction of microRNA/target duplexes. RNA.

[20] Ebert MS, Neilson JR, Sharp PA (2007). MicroRNA sponges: competitive inhibitors of small RNAs in mammalian cells. Nat Methods.

[21] Griffiths-Jones S (2010). miRBase: microRNA sequences and annotation. Curr Protoc Bioinformatics.

[22] Griffiths-Jones S, Grocock RJ, van Dongen S, Bateman A, Enright AJ (2006). miRBase: microRNA sequences, targets and gene nomenclature. Nucleic Acids Res.

[23] Kang Y, Massague J (2004). Epithelial-mesenchymal transitions: twist in development and metastasis. Cell.

[24] Onder TT, Gupta PB, Mani SA, Yang J, Lander ES, Weinberg RA (2008). Loss of E-cadherin promotes metastasis via multiple downstream transcriptional pathways. Cancer Res.

[25] Tran PT, Shroff EH, Burns TF, Thiyagarajan S, Das ST, Zabuawala T, Chen J, Cho YJ, Luong R, Tamayo P, Salih T, Aziz K, Adam SJ, Vicent S, Nielsen CH, Withofs N (2012). Twist1 suppresses senescence programs and thereby accelerates and maintains mutant Kras-induced lung tumorigenesis. PLoS Genet.

[26] Yang MH, Wu MZ, Chiou SH, Chen PM, Chang SY, Liu CJ, Teng SC, Wu KJ (2008). Direct regulation of TWIST by HIF-1alpha promotes metastasis. Nat Cell Biol.

[27] Antonov AV, Knight RA, Melino G, Barlev NA, Tsvetkov PO (2013). MIRUMIR: an online tool to test microRNAs as biomarkers to predict survival in cancer using multiple clinical data sets. Cell Death Differ.

[28] Tian Y, Luo A, Cai Y, Su Q, Ding F, Chen H, Liu Z (2010). MicroRNA-10b promotes migration and invasion through KLF4 in human esophageal cancer cell lines. J Biol Chem.

[29] Moriarty CH, Pursell B, Mercurio AM (2010). miR-10b targets Tiam1: implications for Rac activation and carcinoma migration. J Biol Chem.

[30] Volinia S, Galasso M, Costinean S, Tagliavini L, Gamberoni G, Drusco A, Marchesini J, Mascellani N, Sana ME, Abu Jarour R, Desponts C, Teitell M, Baffa R, Aqeilan R, Iorio MV, Taccioli C (2010). Reprogramming of miRNA networks in cancer and leukemia. Genome Res.

[31] Huang Q, Gumireddy K, Schrier M, le Sage C, Nagel R, Nair S, Egan DA, Li A, Huang G, Klein-Szanto AJ, Gimotty PA, Katsaros D, Coukos G, Zhang L, Pure E, Agami R (2008). The microRNAs miR-373 and miR-520c promote tumour invasion and metastasis. Nat Cell Biol.

[32] Fang L, Du WW, Yang W, Rutnam ZJ, Peng C, Li H, O'Malley YQ, Askeland RW, Sugg S, Liu M, Mehta T, Deng Z, Yang BB (2012). MiR-93 enhances angiogenesis and metastasis by targeting LATS2. Cell Cycle.

[33] Ma L (2010). Role of miR-10b in breast cancer metastasis. Breast Cancer Res.

[34] Miura N, Sato R, Tsukamoto T, Shimizu M, Kabashima H, Takeda M, Takahashi S, Harada T, West JE, Drabkin H, Mejia JE, Shiota G, Murawaki Y, Virmani A, Gazdar AF, Oshimura M (2009). A noncoding RNA gene on chromosome 10p15.3 may function upstream of hTERT. BMC Mol Biol.

[35] Tsuno S, Wang X, Shomori K, Hasegawa J, Miura N (2014). Hsa-miR-520d induces hepatoma cells to form normal liver tissues via a stemness-mediated process. Sci Rep.

