# Supplementary material for: MiR-520d-5p directly targets TWIST1 and downregulates the metastamiR miR-10b
Source: Oncotarget. 2014 Nov 7;5(23):12141–50. doi: 10.18632/oncotarget.2559 (PMC4323010; doi:10.18632/oncotarget.2559)
Supplement: Supplementary file 1 [file oncotarget-05-12141-s001.pdf]

SUPPLEMENTARY FIGURE

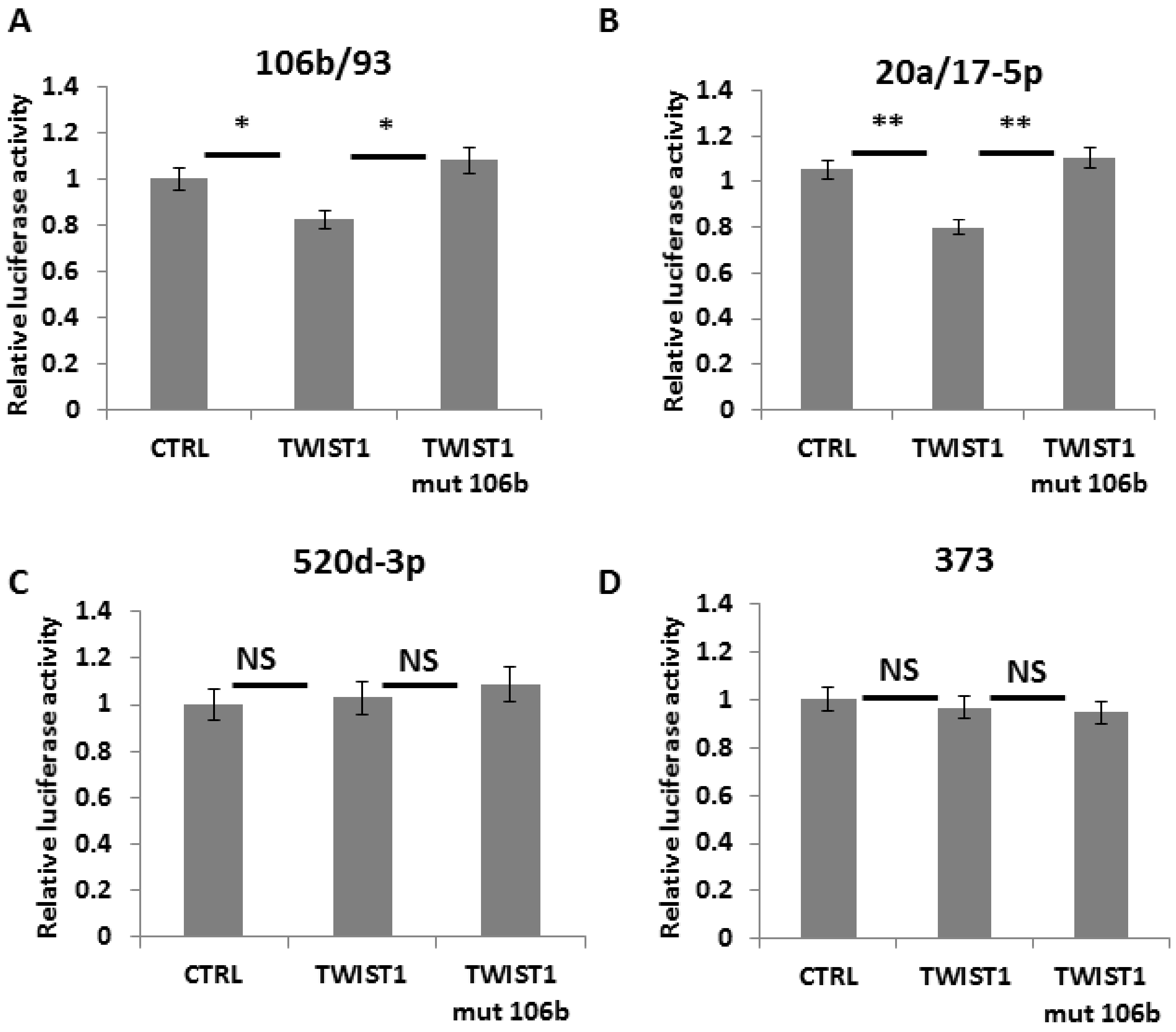

Supplementary Figure S1: miR-106b/93 and miR-17-5p/20a target TWIST1 at the predicted site. Control 3′UTR, WT TWIST1 3′UTR or mutated TWIST1 3′UTR (Mut) were fused to firefly luciferase, and co-transfected with renilla luciferase into DU 145 cells overexpressing miR-93 and miR-106b **(A)**, DU 145 cells overexpressing miR-17-5p and miR-20a **(B)** or DU 145 expressing irrelevant MICB targeting miRNAs miR-520d-3p **(C)** and miR-373 **(D)**.The luciferase activity was measured and normalized to renilla activity. The control 3′UTR activity was set to 1. $*P < 0.045$, $** < 0.02$.
